# Degradable emulsion as vaccine adjuvant reshapes antigen-specific immunity and thereby ameliorates vaccine efficacy

**DOI:** 10.1038/srep36732

**Published:** 2016-11-09

**Authors:** Chung-Hsiung Huang, Chiung-Yi Huang, Chih-Ping Cheng, Shih-Hsiung Dai, Hsin-Wei Chen, Chih-Hsiang Leng, Pele Chong, Shih-Jen Liu, Ming-Hsi Huang

**Affiliations:** 1 National Institute of Infectious Diseases and Vaccinology, National Health Research Institutes, Miaoli 35053, Taiwan; 2 Graduate Institute of Immunology, China Medical University, Taichung 40402, Taiwan

## Abstract

This study describes the feasibility and adjuvant mechanism of a degradable emulsion for tuning adaptive immune responses to a vaccine antigen. We featured a mouse model with ovalbumin (OVA) as the antigen to deepen our understanding of the properties of a degradable emulsion-based adjuvant, dubbed PELC, interacting with immune cells and to elucidate their roles in vaccine immunogenicity *in vivo*. First, we demonstrated that the emulsion, which is stabilized by an amphiphilic bioresorbable polymer, shows degradation in mimic human body conditions and considerable tolerance *in vivo*. Then, we confirmed the model protein could be loaded into the emulsion and released from the matrix in a sustained manner, subsequently driving the production of antigen-specific antibodies. We also comprehended that PELC not only recruits antigen-presenting cells (APCs) to the injection site but also induces the activation of the recruited APCs and migration to the draining lymph nodes. As an adjuvant for cancer immunotherapy, PELC-formulated OVA could strongly enhance antigen-specific T-cell responses as well as anti-tumor ability with respected to non-formulated OVA, using OVA protein/EG7 cells as a tumor antigen/tumor cell model. Accordingly, our data paved the way for the clinical application of degradable emulsions based on amphiphilic bioresorbable polymers as vaccine adjuvants.

Oil-in-water (O/W) emulsions are oil droplets dispersed into a continuous water phase^1^,^2^,^3^. In vaccine formulation, O/W was successfully applied as vaccine adjuvants (from the Latin *adjuvare*, meaning “to help”) for increasing the antigen-sparing efficacy in pandemic influenza vaccine preparedness and for enhancing the immunogenicity of seasonal influenza vaccines in elderly subjects^4^,^5^. In contrast to water-in-oil (W/O) emulsions, which trap the antigenic solution within a continuous oil phase, O/W does not create an antigen vehicle formation^5^; therefore, the mechanisms of action for the enhancement of vaccine immunogenicity are ambiguous.

Our research group was the pioneer who introduces the concepts of “stable” and “degradable” into vaccine emulsions (Fig. 1A). This idea is based on the use of amphiphilic bioresorbable polymers as hydrophilic emulsifiers to stabilize the aqueous/oily interfaces, rendering an emulsion with oil droplets dispersed into the aqueous solution^6^,^7^,^8^,^9^. We proposed that the polymeric emulsifier can stabilize the emulsion during preparation and storage^6^,^7^,^8^; on the other hand, its degradable nature predominates that the emulsion can be destroyed and further to be absorbed post-vaccination^9^. Among these, we optimized a ready-to-use emulsion adjuvant, which dubbed PELC, comprised of phosphate-buffered saline (PBS), squalene oil, the lipophilic emulsifier Span^®^85 (sorbitan trioleate), and the bioresorbable poly(ethylene glycol)-block-poly(lactide-co-ε-caprolactone) (PEG-b-PLACL)^10^. Prior to vaccination, the vaccine antigen was simply mixed with PELC volume/volume to render homogeneous fluid vaccines. Immunogenicity studies in mice demonstrated the potential of the PELC emulsion particles as adjuvants for the development of prophylactic vaccines against emerging infectious diseases, especially influenza-associated illness^10^,^11^, hand-foot-mouth disease^12^, and dengue fever^13^. We also extended these aspects by investigating their sustained delivery against pathogen-associated cancers^14^. In spite of what has been achieved so far, a complete understanding of how degradable emulsions interact with immune cells remains unclear. For vaccination feasibility studies, it is important to evaluate whether PELC emulsion particles can function as a depot formation to ensure the immunoavailability of the antigen *in vivo*. Moreover, understanding the degradation characteristics of the emulsion after vaccination is useful to elucidate the biological interactions as well as the mechanism of action for the targeted delivery. It would be also of great interest to know whether the PELC emulsion can bypass immune tolerance to antigens and strengthen cell-mediated immunity so as to ameliorate the vaccine efficacy.

In the present study, we used a mouse model with ovalbumin (OVA) as the antigen to deepen our understanding of the properties of degradable emulsion-based adjuvants interacting with immune cells and to elucidate their roles in vaccine immunogenicity *in vivo* (Fig. 1B). First, we investigated the relationship between the emulsifier degradation and phase separation of the emulsion. Then, we used fluorescent-labeled antigen components and studied the biodistribution of the PELC-carried antigen. Subsequently, the immunological properties of these formulations were tested by assessing their ability to drive humoral immunity. In parallel, the underlying influence on immune cell recruitment, antigen-presenting cell (APC) activation, T cell differentiation and functionality and the expression of specific key transcription factors were characterized. Finally, the vaccine efficacy was assessed and the respective mode of action was proposed.

## Results

### Chain scission of PEG-b-PLACL dominates the stability of PELC emulsion

The bioresorbable emulsifier used here has initial molecular characteristics of 80 wt% of hydrophilic block PEG and 20 wt% of lipophilic block PLACL with GPC molecular weight of 5,700 Da and polydispersity of 1.1. GPC was used to monitor the molecular weight changes of the polymer during degradation (Figure S1, Supplementary Information). As shown in Fig. 2A, the number-average molecular weight (*M*_n_) of PEG-b-PLACL decreased rapidly during the early stages after the polymer was immersed in water at 37 °C, indicating the random chain scission by hydrolytic cleavage of the PLACL polyesters. The degree of degradation (*M*_n,t_/*M*_n,0_), which was calculated from the ratio of *M*_n_ at each degradation time point to the initial *M*_n_, decreased from the original polymer chain to 0.90 at week 2 and 0.87 at week 4. Afterwards, the degradation rate slowed down. *M*_n,t_/*M*_n,0_ values of 0.83 and 0.79 were detected at weeks 9 and 24, respectively. It should be noted that 20 wt% of degradable PLACL block was contained within the PEG-b-PLACL main chain polymer. These data showed that PEG-b-PLACL started to lose the PLACL moiety after 9 weeks. Because the interface between squalene oil and the PBS aqueous solution was stabilized by the PEG-b-PLACL polymer, we investigated the stability changes in the PELC emulsion at 4 °C and 37 °C over time. The visual aspects of the emulsion remained unchanged for 52 weeks’ storage at 4 °C. At 37 °C, an isotropic emulsion was stored for at least 6 months; beyond 30 weeks, about 10% of free oil at the surface layer was dissociated from the emulsion (Fig. 2B). Dynamic light scattering (DLS) showed that the emulsified PELC particles possessed a unimodal distribution, with an average diameter of approximately 500 nm. Beyond 30 weeks’ storage at 37 °C, the particle size increased due to the disassociation of the immiscible squalene and water.

### Degradable emulsion increases the immunoavailability of vaccine antigen

We exploited the ability of emulsified particles to serve as either carriers or vehicles to deliver biologically active agents (e.g., antigens and immunostimulatory adjuvants) to immune cells in a targeted and prolonged manner, thereby effectively probing and boosting the vaccine’s immunogenicity. To this, we determined whether the model protein antigens could be loaded into the emulsified formulation and released from the matrix in a sustained manner *in vivo*. Mice were vaccinated i.m. with 10 μg of OVA-Alexa 647 (Alexa Fluor^®^647-conjugated ovalbumin protein) in both quadriceps with either antigen in PBS or adjuvanted with PELC. As shown in Fig. 3A, the fluorescence signal was clearly induced in the quadriceps muscles of the mice at 0 h post-injection. The signal dropped dramatically in the OVA-Alexa 647-treated mice within 24 h. No signals characteristic of Alexa Fluor^®^ were detected on the IVIS spectra beyond 72 h, indicating the absence of OVA-Alexa 647. In contrast, the signal gradually decreased in the PELC-adjuvanted OVA-Alexa 647-injected mice and returned to baseline within approximately 336 h after injection. The OVA-Alexa 647 fluorescence signals at the injection sites were strongly influenced by the presence of the PELC emulsion, suggesting that PELC was a good candidate for the prolonged release of the hydrophilic model antigen protein OVA.

### Degradable emulsion prolongs antigen-specific humoral immunity

Following vaccination, vaccine antigens may directly act on the APCs for integrating the whole immune responses, bind to the B cells for generating the antigen-specific antibodies, or undergo degradation^8^. Ideally, a single injection can achieve a prime-boost vaccination with the aid of a stepwise release of vaccine antigens. We hypothesized that the production of antigen-specific antibodies could be driven by the sustained release of the antigen protein. The results of the antigen-specific antibody production analysis are shown in Fig. 3B. Following a single-dose i.m. injection, the sera from mice vaccinated with OVA alone elicited OVA-specific IgG antibodies with geometric mean titers (GMT) less than 1,000 at week 4. The titer increased slightly at week 8 and then fluctuated. When the same amount of OVA was co-administered with PELC, the induced OVA-specific IgG titers were strongly increased compared to those induced by non-adjuvanted OVA. The IgG titers peaked at week 8 and lasted for at least 20 weeks, indicating the improvement of the longevity of the induced antibody response when the antigen was adjuvanted with the emulsion. The IVIS imaging and antigen-specific antibody response data indicated that the PELC emulsion provided an antigen depot for the sustained release of antigen protein. Because the host immune cells (APCs and B cells) are continuously exposed to antigen, PELC allows the host to integrate a longer duration of immune responses compared to non-adjuvanted OVA.

Histological examination and serum biochemistry test for the immunized mice were assessed to monitor safety aspects and biocompatibility of PELC (Figure S2, Supplementary Information). Overall speaking, injection with PELC induced less tissue damage compared to incomplete Freund’s adjuvant (IFA, W/O emulsion). Notably, cells infiltrated all around the vacuoles (identified as PELC emulsion) at the injection sites within 5 weeks post-injection, and were retained for up to 10 weeks (Figure S3, Supplementary Information). The vacuoles became smaller over time, showing PELC absorption *in vivo*. The data showed that the presence of PELC emulsion particles in the vaccine formulation really enhances the immunogenicity of vaccine antigen without alternating the safety aspects, which is a feature of great interest for further *in vivo* application.

### Degradable emulsion elicits appropriate cell-mediated immunity

The cell-mediated immunity plays a crucial role in removing virus-infected cells and in defending against cancers. APCs capture antigens in peripheral tissues and then migrate into the draining lymph nodes (LNs) for antigen presentation to lymphocytes^15^. After antigen uptake, APCs process the protein antigens into peptide epitopes for presentation to T cells and elicit antigen-specific immune responses by regulating costimulatory signals or major histocompatibility complex (MHC) expression^16^. To study the impact of degradable emulsion on the recruitment of APCs to the inter-particle matrices, we vaccinated mice through footpad injection with OVA alone or PELC-adjuvanted OVA and then performed a histological examination at the injection sites at the designated time points. Figure 4A shows the immunohistochemical staining (IHC) images of CD11b/c^+^ cells, which are commonly identified as the most potent APCs, at the injection site after injection with OVA alone or OVA formulated with the PELC emulsion. Very few CD11b/c^+^ cells (brown signals around blue nuclei) were observed in the mice injected with OVA alone compared to the mice injected with PELC-adjuvanted OVA, indicating that only OVA formulated with PELC recruited an influx of CD11b/c^+^ cells to the injection site. This finding is consistent with reports in the literature that the squalene-in-water emulsion, MF59, drives the recruitment of CD11b^+^MHCII^+^ cells from the bloodstream into the muscle after intramuscular injection^16^. Next, we analyzed the activation of CD11b^+^ cells and CD11c^+^ cells in lymphoid cells. As shown in Fig. 4B, vaccination with PELC-adjuvanted OVA increased the expression of MHC II and costimulatory molecule CD40, which are commonly identified activation markers for APCs, on CD11b^+^ cells day 7 post-injection. Similar results were observed with CD11c^+^ cells post-injection. Based on above results, PELC not only recruits APCs to the injection site but also induces the activation of the recruited APCs and migration to the LNs.

The antigen-loaded APCs then fragment the antigen into antigenic peptides for presentation to T cells and elicit antigen-specific immune responses by inducing the differentiation and activation of the T cell populations, such as CD4^+^ T helper (T_H_) cell, regulatory T (T_reg_) cell and CD8^+^ cytotoxic T lymphocyte (CTL) subsets^17^. Therefore, the phenotype, differentiation and functionality of the T cell subsets were comprehensively investigated by analyzing surface marker, transcription factor and cytokine expression in splenocytes. As shown in Fig. 4C, mice vaccinated with PELC-adjuvanted OVA exhibited high levels of IFN-γ and IL-2 (the major T_H_1-type cytokines) and IL-17 (the major T_H_17-type cytokine) but a low level of IL-10 (the major T_H_2- and T_reg_-type cytokine) secretion by OVA-restimulated splenocytes compared to mice vaccinated with OVA alone. The concentration of IL-4 (the major T_H_2-type cytokine) was at an undetectable level. To extend our analysis of antigen-specific T cell differentiation and functionality, each CD4^+^ T cell lineage was analyzed based on the mRNA expression of the specific key transcription factors T-bet (T_H_1), GATA3 (T_H_2), RORγt (T_H_17) and Foxp3 (T_reg_)^18^,^19^. Vaccination with PELC-adjuvanted OVA augmented T-bet and RORγt mRNA expression compared to vaccination with OVA alone (Fig. 4D); however, the mRNA level of GATA3 and Foxp3 were either not expressed or were no different from non-adjuvanted OVA control, suggesting that the PELC emulsion may be a potential immunomodulatory agent that reshapes both antigen-specific humoral and cellular immunity. Because CTLs played a critical role in removing virus-infected cells and in defending against cancers, we studied the phenotype and functional activation marker of CTLs, called CD107a^20^. As shown by flow cytometric analysis (Fig. 4E), vaccination with OVA alone led to minimal antigen-specific CTL activation, as expected. The mean fluorescence intensity (MFI) of CD107a on CD8^+^ T cells was increased from 136 ± 9 of non-adjuvanted OVA to 176 ± 6 of PELC-adjuvanted OVA. These results substantiated that the PELC emulsion is capable of driving high numbers of CD8^+^CD107a^+^ cells in the spleens of vaccinated mice. Together, PELC emulsion has the intermediate mechanisms between the particulate depot and immunomodulator in terms of magnitude of the induced activation of lymphoid APCs and splenic T cells, compared with O/W and W/O emulsions (Figures S4 to S6, Supplementary Information).

### Degradable emulsion ameliorates vaccine efficacy

To evaluate whether the reshaped cell-mediated immunity reflected antigen-specific cytotoxicity *in vivo*, we applied a cancer immunotherapy consisting of OVA protein/EG7 cells (a thymoma cell line stably transfected with OVA complementary DNA) as a tumor antigen/tumor cell model^21^. As shown in Fig. 5A, the tumors grew progressively and lethality appeared in the mice that did not receive any treatment (PBS control group) within 30 days. Similarly, no protection was observed for the mice that received OVA alone, and all mice succumbed before day 60. Interestingly, a smaller tumor volume and higher survival rate were observed in mice vaccinated with PELC-adjuvanted OVA compared to mice vaccinated with non-adjuvanted OVA, indicating that PELC provided a better protective capacity. We also measured the secretion of IFN-γ and IL-17 by tumor-infiltrating lymphocytes. As shown in Fig. 5B, tumor-bearing mice vaccinated with PELC-adjuvanted OVA elicited a high number of both IFN-γ^+^ and IL-17^+^ cells at tumor-adjacent counterparts compared to non-adjuvanted OVA. These results demonstrate that PELC is a promising and effective adjuvant for an antitumor immunotherapeutic vaccine.

## Discussion

Conventionally, the O/W interface is stabilized by a hydrophilic emulsifier, e.g. ethoxylated sorbitan fatty acid esters (known as Tween^®^). However, it has been well documented that Tween^®^ series may cause toxicities including severe non-immunological anaphylactoid reactions^6^,^7^. Here we use PEG-b-PLACL/Span^®^85 as surface-active components to stabilize oil/water interfaces, rendering emulsified PELC particles. In this form, PELC particles can act as a sustained-release depot for vaccine antigens. Since polymers derived from lactide and ε-caprolactone have been widely considered as degradable polyester, the hypothesis was that loss of PLACL moiety of the amphiphilic bioresorbable polymer PEG-b-PLACL may affect the stability of the emulsion, leading to phase separation of oil/water and further absorption *in vivo*. The findings of our previous work showed that the degradation of the emulsifier poly(ethylene glycol)-block-polylactide (PEG-b-PLA) directly affected the stability of the PEG-b-PLA-stabilized squalane-in-water emulsion^9^. In this study, hysteresis happens between the degradation of emulsifier PEG-b-PLACL and the stability of emulsion PELC (Fig. 2). This finding was assigned to the fact that the presence of excipient Span^®^85 in the oily phase may not only play an important role in governing the dispersion properties of the emulsion but also provide a potential way to stabilize PELC particles^7^. However, these parameters are strongly influenced by an optimization of the emulsification process and the surfactant system.

Many studies have attempted to elicit anti-tumor immune responses based on recombinant tumor-associated antigen (TAA) vaccines for the treatment of cancers^22^. However, vaccination with TAA alone is not immunogenic due to self-tolerance^20^,^21^,^22^. Regarding this, the development of an adjuvant with a high potential to enhance cellular responses is key to improving the prospects of TAA-based cancer vaccines^21^,^22^. Positive results have been obtained with CIMAvax-EGF for the treatment of non-small-cell lung cancer carcinoma, which consists of a human-recombinant epidermal growth factor (EGF) linked to a carrier protein and Montanide ISA 51 (a W/O emulsion) as adjuvant^23^,^24^. Although CIMAvax-EGF works well by inducing a patient’s body to release antibodies against EGF, repeated dose intramuscular injection of this vaccine produced local damage at the injection site^24^. To ameliorate the reactogenicity profiles and also to improve the injectability, O/W emulsions provide a better-adapted vaccination strategy in which low oil content within when performing vaccination^5^. Besides destroying the tumor cells, T-cell immunity is also regarded as crucial in clearing virus-infected cells. It is commonly believed that protective immunity against influenza virus infection is mediated by neutralizing antibodies, whereas T cells may limit the severity of influenza associated illness by new strains in the absence of specific antibody responses^25^. There is evidence that T cells cannot prevent virus infection but they can sense infected cells by recognizing epitopes of viral protein complexed to human leukocyte antigen (HLA) molecules on the surface of infected epithelial cells or APCs^25^. Effective vaccination was also correlated with the induction of the T_H_1 cytokines (including IFN-γ), especially in the elderly who undergo relative reduction in CTL activity as they age^26^. Therefore, increasing IFN-γ induction as well as CTL activity via vaccination is thought to be an important strategy for overcoming the age-related influenza susceptibility. In fact, a seasonal influenza vaccine formulated with the O/W adjuvant has already approved in humans for the prevention of seasonal influenza in people 65 years of age and older^5^,^27^. Nevertheless, no cancer therapeutic vaccine formulated with this type of emulsions was registered for human use. Our results suggest that PELC may be a potential adjuvant to modulate/regulate the immune responses (Figs 3 and 4), the development of PELC in the present study allows of optimization of a process for the design and fabrication of vaccine formulations in specific applications.

**Figure 1:**
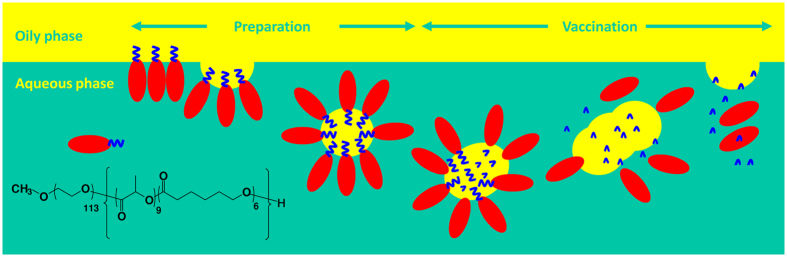
Schematic representation of a degradable emulsion stabilized by bioresorbable polymers. The polymeric emulsifier stabilizes the emulsion during preparation and storage; however, its degradable nature can destroy the emulsion post-vaccination.

**Figure 2:**
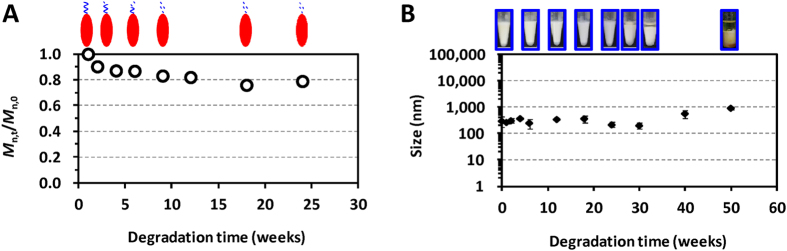
Characteristic changes of PEG-b-PLACL and PEG-b-PLACL-stabilized squalene emulsion at 37 °C. (**A**) *In vitro* hydrolytic degradation of PEG-b-PLACL performed in water at 37 °C. *M*_n,t_/*M*_n,0_ was calculated from the ratio of the number-average molecular weight (*M*_n_) of the polymer at each degradation time point to the initial *M*_n_. (**B**) Visual aspects of the emulsion upon storage at 37 °C for one year. The particle size of the emulsified formulations was measured by DLS. The data are expressed as the mean value with standard deviation of three samples.

**Figure 3:**
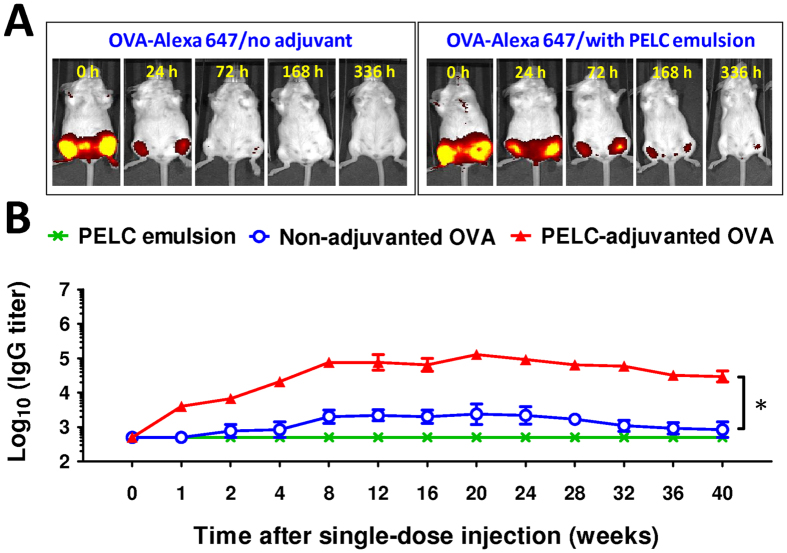
Degradable emulsion prolongs antigen retention and strengthens antigen-specific humoral immunity *in vivo*. (**A**) *In vivo* distribution of antigen. The mice were vaccinated with 10 μg of OVA-Alexa 647 with or without PELC emulsion in both quadriceps and monitored by In-life fluorescence image system. (**B**) Effect of PELC emulsion on IgG antibody titers against OVA in mice (6 mice/group) vaccinated with PELC alone, OVA alone or PELC-adjuvanted OVA. The data are expressed as the GMT ± standard deviation. *P < 0.05.

**Figure 4:**
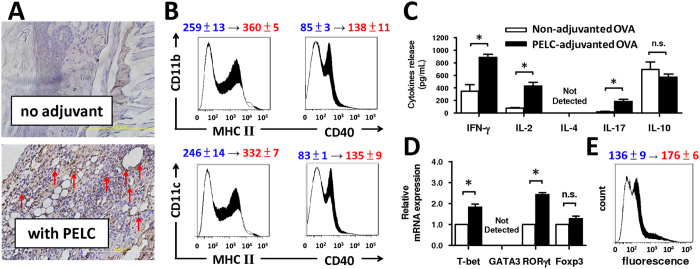
Degradable emulsion recruits and activates APCs, and tunes cell-mediated immunity. Three mice per group were injected once s.c. in both hind footpads with 10 μg/mL of OVA alone or adjuvanted with PELC. (**A**) IHC images of the injection site at day 7 post-vaccination (original magnification, ×400). Cells with brown signals around the blue nuclei are CD11b/c^+^ cells (red arrow). (**B**) The draining LN cells were harvested on day 7, and the expression levels of MHC class II and CD40 were determined by flow cytometry. The data shown were gated on CD11b^+^ or CD11c^+^ cells. Seven days post-vaccination, the splenocyte suspensions (5 × 10^6^ cells/mL) were pooled and incubated in the absence or presence of OVA protein (50 μg/mL) for 72 h. (**C**) Supernatants from triplicate cultures were collected to measure the concentrations of cytokines IFN-γ, IL-2, IL-17 and IL-10 by ELISA via paired antibodies. (**D**) The mRNA expression levels of T-bet, GATA3, RORγt and Foxp3 were measured by RT-PCR. The data are normalized to the β-actin mRNA and graphed as the fold change over the non-adjuvanted OVA control. *P < 0.05. n.s.: no significant difference. (**E**) The MFIs of CD107a on CD8^+^ T cells were determined by flow cytometry. The change in the mean fluorescence intensity from non-adjuvanted OVA to PELC-adjuvanted OVA is indicated. White area: non-adjuvanted OVA; black area: PELC-adjuvanted OVA. The data are expressed as the mean ± standard deviation of four independent experiments.

**Figure 5:**
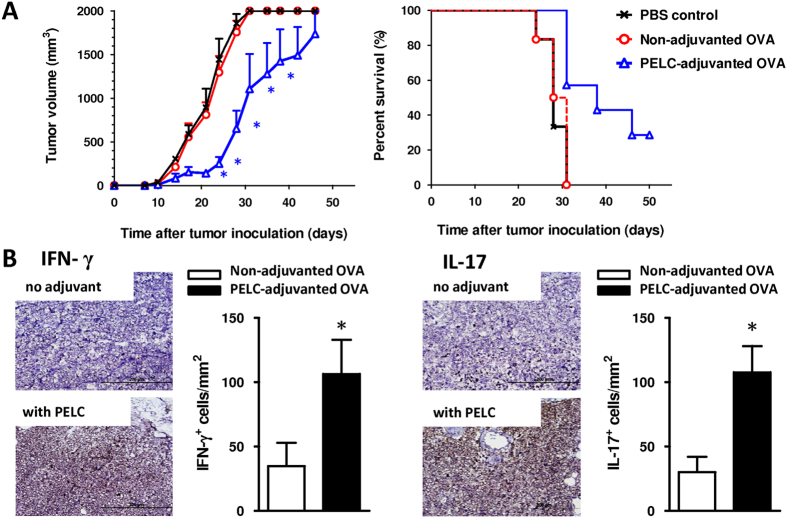
Immunotherapeutic effect of the OVA protein adjuvanted with PELC and administered to C57BL/6 mice bearing EG7 tumor cells. (**A**) Anti-tumor efficacy; (**B**) IHC analysis of IFN-γ and IL-17 expression at tumor-adjacent counterparts. Mice were inoculated s.c. in the flank with EG7 tumor cells (2 × 10^5^ cells/mouse). Upon the appearance of palpable tumors, six mice per group were injected s.c. at the tail base with 10 μg/dose OVA protein with or without PELC on day 7. Mice were euthanized when the tumor volume exceeded 2,000 mm^3^. The data are expressed as the mean value ± standard deviation. The tumor volumes were compared on days 21, 24, 28, 31 and 35 following the onset of tumor growth in the vaccine group. On day 31 after tumor cell inoculation, tissue sections from tumor-adjacent counterparts (n = 3) were IHC stained with IFN-γ and IL-17 antibodies (original magnification, ×400). Cells with brown signals around the blue nuclei indicate IFN-γ^+^ or IL-17^+^ cells. Statistical significance was determined by performing the two-tailed Student’s *t*-test. *P < 0.05 compared with the non-adjuvanted OVA group. The data are representative of two independent experiments.

Figure 1 summarizes the respective mechanism by which a degradable emulsion can reshape the immunogenicity profiles and ameliorate the vaccine efficacy. During manufacturing and storage, the metabolizable oil (squalene) was well-dispersed in the aqueous solution by the amphiphilic bioresorbable polymer. Following vaccination, the emulsified particles functioned as an antigen depot formation and were targeted by APCs. The emulsion shows degradation and absorption *in vivo*. Then, the APCs were subsequently activated and migrated to the draining lymph nodes, where the antigen-loaded APCs processed the antigen into peptide epitopes for presentation to CD8^+^ CTLs and CD4^+^ T_H_ cells through the MHC pathways. The activated APCs induced by the degradable emulsion promoted CTL activation and T_H_1 and T_H_17 cell polarization. Conversely, T_reg_ cell differentiation was attenuated. Note that the interaction between CD4^+^ T_H_ cells and B cells was also necessary to generate antibodies, leading to phagocytosis, complement activation and antibody-dependent cellular cytotoxicity, which are critical mechanisms for the destruction of tumor cells as well as removing virus-infected cells^22^. Accordingly, the growth of the tumor and the mortality of the tumor-bearing mice were suppressed by the aid of degradable emulsion, offering the possibility of complementary enhancement for the development of novel antigen delivery systems for vaccine adjuvants and immunotherapy technologies.

In this study, we reported the mechanisms underlying degradable emulsion-induced immune responses. We demonstrated that emulsion made from bioresorbable polymers shows degradation and considerable tolerance *in vivo*. As vaccine adjuvant, it prolongs antigen retention, recruits and activates APCs, and strengthens both antigen-specific humoral and cellular immunity, thereby ameliorating the vaccine efficacy. The information gathered from murine studies will enhance our efforts to deepen our understanding of the role of degradable emulsion vaccine formulations in vaccine immunogenicity. Further investigations are under way to combine the degradable emulsion with potential immunostimulatory molecules to prolong the survival of tumor-bearing mice.

## Materials and Methods

### Reagents, antibodies and cell lines

The AB-type diblock copolymer PEG-b-PLACL was synthesized by ring-opening polymerization of DL-lactide and ε-caprolactone in the presence of polyethylene glycol 5,000 monomethyl ether and SnOct_2_^6^,^7^. Ovalbumin (OVA, Grade V) was purchased from Sigma-Aldrich (MO, USA). The ELISA reagents and standard were purchased from R&D systems, Inc. (MN, USA). The 10% neutral buffered formalin and decalcifying solution were purchased from Leica Biosystems Richmond Inc. (IL, USA). Trilogy, 3,3′-diaminobenzidine (DAB) and the other reagents used for IHC staining were purchased from Cell Marque Corporation (CA, USA), Thermo Fisher Scientific, Inc. (CA, USA) and Nichirei Bioscience Inc. (Tokyo, Japan), respectively. The antibodies used for IHC staining and flow-cytometry analysis were purchased from BioLegend Inc. (CA, USA). The reagents used for RT-PCR were purchased from iNtRON Biotechnology Inc. (Kyungki-Do, Korea). The EG7 cell line (American Type Culture Collection, CRL-2113), which is a stable transfectant of the murine OVA-expressing EL4 thymoma (H-2b), was maintained in complete RPMI 1640 medium supplemented with G418 (0.4 mg/mL; Calbiochem^®^, Merck Millipore, Darmstadt, Germany) for the tumor challenge study.

### Degradation of PEG-b-PLACL

A total of 10 mg of PEG-b-PLACL was dissolved in an Eppendorf tube containing 100 μL of distilled deionized water. The tubes were placed in a circulating water bath at 37 °C. At predetermined time points, the specimens were collected and lyophilized before being subjected to gel permeation chromatography (GPC) analysis. GPC was performed using a setting composed of an isocratic pump, a refractive index detector, and two size exclusion columns connected in series: one PLgel 5 μm guard column (7.5 × 50 mm) and one PLgel 5 μm mixed-D column (7.5 × 300 mm). The mobile phase was tetrahydrofuran (THF), and the flow rate was 1.0 mL/min. The data were expressed with respect to the polystyrene standards (Varian, Inc., Amherst, MA, USA).

### Adjuvant preparation

For PELC preparation, 120 mg of PEG-b-PLACL, 0.8 mL of PBS, and 1.1 mL of an oily solution consisting of squalene and Span^®^85 (85/15 v/v) were emulsified using a homogenizer at 6 000 rpm for 5 min. The stability of emulsions was recorded at 4 °C and 37 °C by visual observation. The PELC-formulated vaccine was investigated by re-dispersing the stock emulsion into the bulk vaccine and mixing with a test-tube rotator at 5 rpm for at least 1 h prior to vaccination. The particle size was measured by dynamic light scattering (DLS) technique (Brookhaven 90 plus particle size analyser, Brookhaven Instruments Limited, NY, USA).

### Mice and ethics statement

Six-to-eight-week-old female BALB/c and C57BL/6 mice were obtained from the National Laboratory Animal Center. All mice were housed at the Laboratory Animal Facility of the NHRI, Miaoli County, Taiwan. All experiments were conducted in accordance with the guidelines of Laboratory Animal Center of NHRI. All animal studies were approved by the NHRI Institutional Animal Care and Use Committee (NHRI-IACUC-103027-AC).

### *In vivo* fluorescence imaging

Alexa Fluor^®^ 647-conjugated ovalbumin protein (OVA-Alexa 647, absorption 650 nm, fluorescence emission 668 nm) was purchased from Molecular Probes (Eugene, OR, USA). BALB/c mice were injected intramuscularly (i.m.) in both quadriceps muscles with 5 μg of OVA-Alexa 647 per quadriceps with either with 100 μL of PBS (control group) or supplemented with PELC diluted in PBS (10% v/v, total volume of 100 μL). The mice were anesthetized with isoflurane at a maintenance concentration of 2.5% and oxygen pressurized at 4 kg/cm^2^ using the XGI-8 Anesthesia System. In-life fluorescence analysis was performed at 0, 24, 72, 168, and 336 h post-injection using a Xenogen IVIS^®^ Spectrum 200 Imaging System (Caliper Life Sciences, USA). The fluorescent measurement was quantified using the IVIS Living Image 4.0 software package.

### Vaccination and ELISA immunoassay

BALB/c mice (six mice per group) were injected once i.m. with 10 μg of OVA (non-adjuvanted or adjuvanted with 10% v/v PELC). Serum samples were collected from the vaccinated mice via the submandibular veins, and the presence of specific antibodies in the sera was determined by enzyme-linked immunosorbent assay (ELISA). In brief, 100 μl of diluted OVA (5 μg/mL) was coated in 96-well microtiter plates with 0.05 M carbonate buffer (pH 9.6, Sigma) by overnight incubation at room temperature. The coated plates were washed once with PBS containing 0.05% Tween^®^20 (Sigma) and then blocked with 1% bovine serum albumin (BSA, Sigma) in PBS at room temperature for 2 h. Diluted sera (starting dilution 1:1 000, serial two-fold serum dilutions) from the vaccinated animals were applied to the wells and incubated at room temperature for 2 h. Following the addition of HRP-conjugated goat anti-mouse IgG (1:5 000, ICN Cappel, Aurora, OH, USA), the assay was developed with the tetramethylbenzidine chromogen substrate (NeA-Blue^®^, Clinical Science Products Inc, MA, USA) for 20 min at room temperature in the dark. The plates were read at 450 nm using an ELISA plate reader (Molecular Devices, Sunnyvale, CA, USA). The titers were determined from the reciprocal of the final dilution that gave an optical two-fold absorbance of the pre-immune sera. For calculation purposes, an undetectable level was scored as a titer equal to 500.

### Lymphoid APCs activation

C57BL/6 mice (three mice per group) were injected subcutaneously (s.c.) in the both hind footpads with OVA (10 μg in 100 μL sterilized PBS per mouse) or PELC-adjuvanted OVA (10% v/v PELC of the total volume). Seven days after injection, the footpad tissues were excised, sectioned, and stained with CD11b/c antibodies for immunohistochemical examination. The lymphoid cells from the inguinal and popliteal lymph nodes (LNs) were collected from the vaccinated mice. The cell suspensions were harvested and re-suspended in red blood cell lysing buffer (ACK buffer: 155 mM NH_4_Cl, 10 mM KHCO_3_, and 0.1 mM EDTA) for 1 min. The cells were collected and stained with anti-CD11b, anti-CD11c, anti-CD40 and anti-MHC II antibodies and then subjected to flow-cytometry analysis (LSRII; BD Immunocytometry Systems, CA, USA).

### T-cell investigations

C57BL/6 mice (three mice per group) were injected s.c. in both hind footpads with 10 μg/mice of OVA alone or adjuvanted with 10% v/v PELC. Seven days after injection, the spleens were collected from the vaccinated mice, and 5 × 10^6^ splenocytes were cultured in the absence or presence of OVA (50 μg/mL) for 72 h. The supernatants were collected for IL-2, IL-4, IL-10, IL-17 and IFN-γ measurement by ELISA (DueSet^®^ ELISA Development kit, R&D Systems, Inc., MN USA) following the supplier’s instruction. The splenocytes were stained with anti-CD4, anti-CD8 and anti-CD107a and then subjected to flow cytometry analysis. Total RNA was extracted from the splenocytes in each group using the TRI Reagent (Sigma) according to the manufacturer’s instructions. The steady-state mRNA expression levels of T-bet, GATA3, Foxp3, RORγt and β-actin were measured by reverse transcription-polymerase chain reaction (RT-PCR). All isolated RNA samples were confirmed to be free of DNA contamination by the absence of product after PCR amplification in the absence of reverse transcription. For reverse transcription, 10 μg of total RNA was reverse-transcribed into cDNA using the Maxime RT PreMix Kit (iNtRON Biotechnology, Inc.) and random primers. The reverse transcription proceeded at 45 °C for 60 min and then at 95 °C for 5 min. Next, 2x PCR Master Mix Solution (iNtRON Biotechnology, Inc.) and 10 pmol of each forward and reverse primer specific for the gene of interest were added to each cDNA sample for PCR. The samples were heated to 94 °C for 2 min and cycled 30–37 times at 94 °C for 30 seconds, 55 °C for 45 seconds, and 72 °C for 60 seconds, followed by an additional step at 72 °C for 5 min. The PCR products were electrophoresed in 2% agarose gels and stained with 0.1 μg/mL SYBR^®^ Green (Thermo Fisher Scientific, Inc., CA, USA) for visualization. Quantification was performed by assessing the optical density of the DNA bands using the ImageJ image processing and analysis program (MD, USA). The results are expressed as the density ratio between the gene of interest and the reference standard (β-actin).

### Tumor challenge study

C57BL/6 mice (six mice per group) were first inoculated s.c. with EG7 tumor cells (2 × 10^5^ cells per mouse) in the flank. Upon the appearance of palpable tumors, the mice were injected s.c. at the base of the tail with 10 μg of OVA protein non-adjuvanted or adjuvanted with PELC on day 7. The tumor sizes were measured using a caliper (Digimatic Caliper, Mitutoyo, Japan) in two vertical dimensions twice a week. The tumor volumes were calculated according to the formula: (length × width^2^)/2. The mice were euthanized when the tumor volume exceeded 2,000 mm^3^. Median survival was calculated by the Gehan-Breslow-Wilcoxon method. On day 31 after tumor cell inoculation, three mice per group were euthanized to excise tumor tissues for immunohistochemistry (IHC) staining. The isolated tumor tissues were washed with saline and then fixed in 10% neutral buffered formalin for 24 h. Tissue blocks were embedded in paraffin and cut into 4–5 μm thick sections on silane-coated slides. For de-waxing, the slides were immersed in xylene three times for 5 min and then immersed sequentially in 100%, 95%, 90%, 80%, and 60% ethanol for 5 min each for hydration. The hydrated slides were immersed in Trilogy at 121 °C for 30 min. The slides were treated with 3% H_2_O_2_ for 15 min and blocked with normal horse serum for 1 h. After washing, the slides were incubated with primary antibodies at room temperature in the dark for 1 h and then incubated with the antigen/antibody/Universal Immuno-Peroxidase Polymer complex (Leica Biosystems Richmond Inc., IL, USA) for 1 h. For visualization, the slides were treated with the HRP substrate DAB for 5 min, followed by hematoxylin counterstaining for 5 min. The number of IHC-positive signals was quantified using the ImageJ image processing and analysis program. Three measurements per tissue section and 3–6 sections per group were analyzed at a 400-fold magnification.

### Statistical analysis

The graphs were performed using GraphPad Prism version 5.02 (GraphPad Software, Inc.). The comparison of the survival curve between groups was calculated using the log-rank (Mauted-Cox) test. The comparison of the antibody titers between the PELC-treatment groups and the no adjuvant group was determined by performing the two-tailed Student’s *t*-test on log_10_-transformed values, using Microsoft Excel.

## Additional Information

**How to cite this article**: Huang, C.-H. *et al*. Degradable emulsion as vaccine adjuvant reshapes antigen-specific immunity and thereby ameliorates vaccine efficacy. *Sci. Rep*. **6**, 36732; doi: 10.1038/srep36732 (2016).

**Publisher’s note**: Springer Nature remains neutral with regard to jurisdictional claims in published maps and institutional affiliations.

## Supplementary Material

Supplementary Information
