# Supplementary material for: Degradable emulsion as vaccine adjuvant reshapes antigen-specific immunity and thereby ameliorates vaccine efficacy
Source: Sci Rep. 2016 Nov 9;6:36732. doi: 10.1038/srep36732 (PMC5101498; doi:10.1038/srep36732)
Supplement: Supplementary Information [file srep36732-s1.doc]

Degradable emulsion as vaccine adjuvant reshapes antigen-specific immunity and thereby ameliorates vaccine efficacy

Chung-Hsiung Huang^1,+^, Chiung-Yi Huang^1,+^, Chih-Ping Cheng^1^, Shih-Hsiung Dai^1^, Hsin-Wei Chen^1,2^, Chih-Hsiang Leng^1,2^, Pele Chong^1,2^, Shih-Jen Liu^1,2,*^ & Ming-Hsi Huang^1, 2,*^

^1^National Institute of Infectious Diseases and Vaccinology, National Health Research Institutes, Miaoli 35053, Taiwan

^2^Graduate Institute of Immunology, China Medical University, Taichung 40402, Taiwan

^*^Correspondence: levent@nhri.org.tw (S.-J.L.) or huangminghsi@nhri.org.tw (M.-H.H.).

^+^These authors contributed equally to this work.


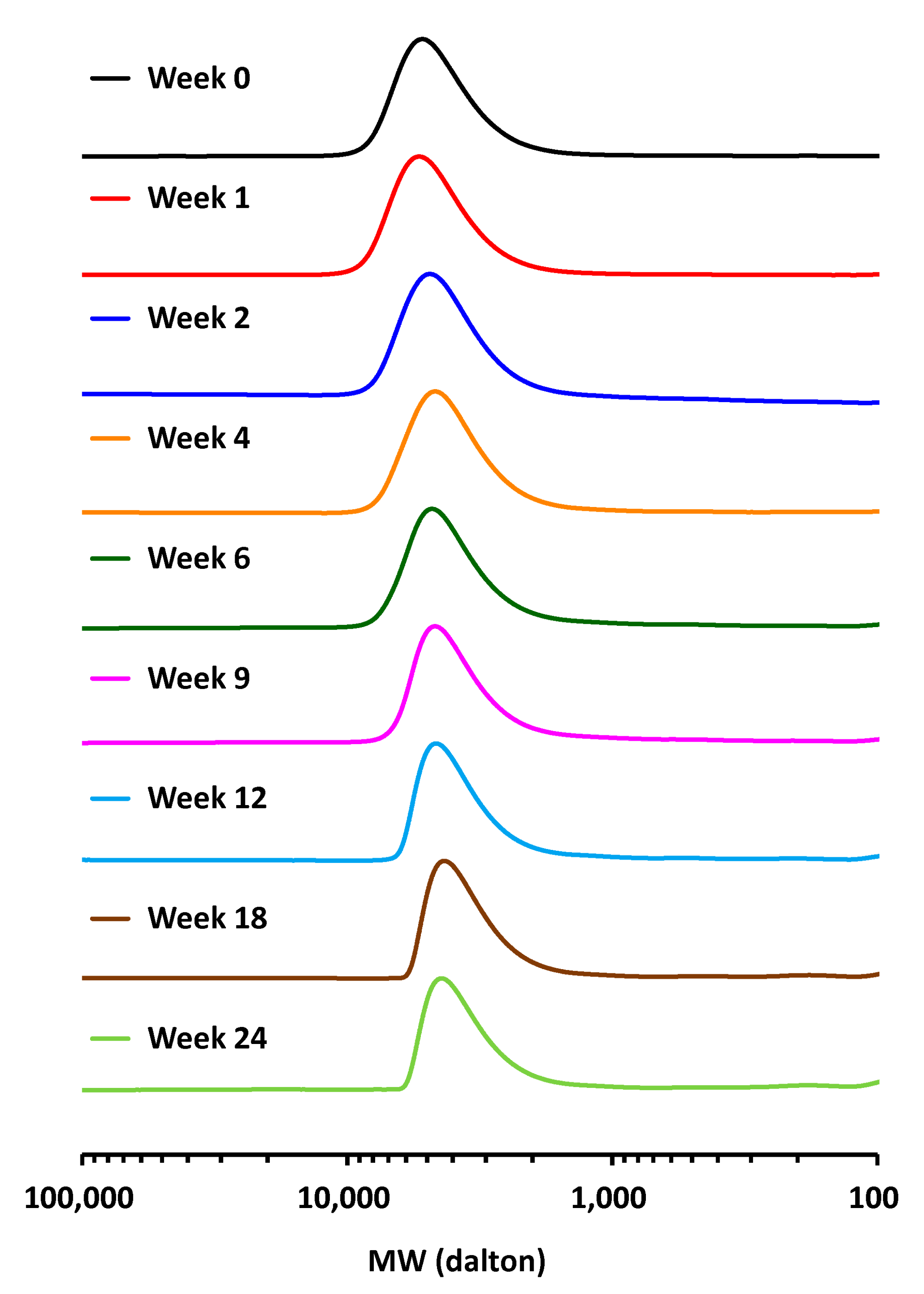


**Figure S1.** MW changes of PEG-b-PLACL during degradation in distilled deionized water at 37°C. Degradation products were recovered by lyophilization and monitored by GPC. Degradation samples were frozen, dried and re-dissolved in THF and applied to GPC with a 1.0 ml/min flow rate. The data were expressed with respect to the polystyrene standards.


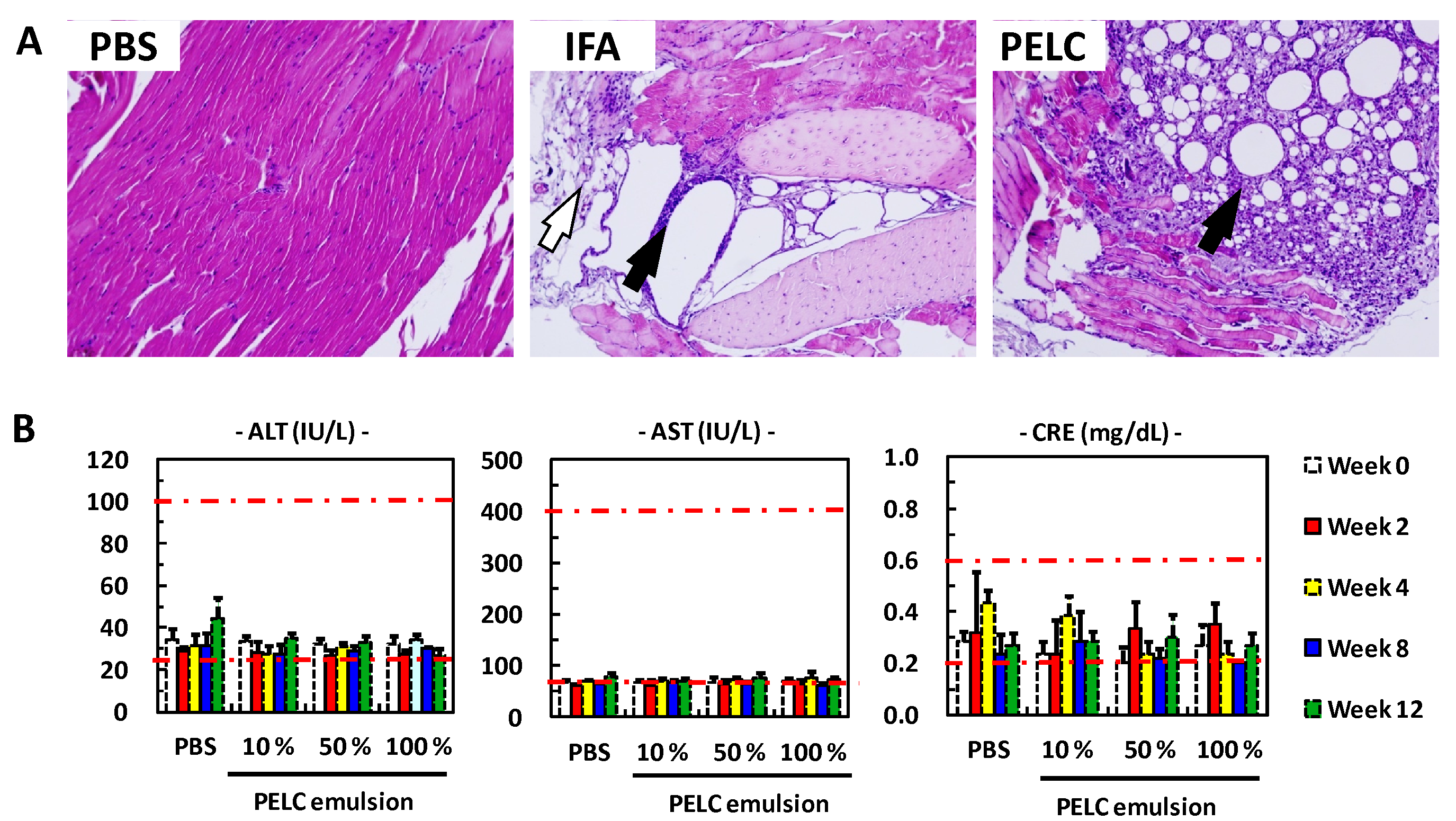


**Figure S2.** *In vivo* biocompatibility of PELC. (A) Histopathological examination of tissue sections at the injection site following a single i.m. injection of mice with PBS; IFA; and 10 % v/v PELC. The muscles at the injection sites were excised, fixed with 10% formaldehyde overnight and embedded in paraffin. Then, 4 μm sections were stained with H&E and examined using an Olympus DP70 microscope (×200 magnification). Inflammation is widespread with the influx of a large number of polymorphonuclear cells (filled arrow) in the PELC and IFA groups, and severe necrosis of the adipose tissue is observed at the local injection site (open arrow) in the IFA control group. (B) Serum AST, ALT, and CRE levels of mice treated with PELC. The mice were injected once i.m. with 0, 10, 50, or 100% v/v PELC diluted in PBS. Serum samples were collected, and the concentrations of the biochemical indicators were measured by colorimetric analysis. The data are presented as the mean ± standard deviation of five mice per group. The dotted horizontal lines represent the normal ranges in mice.


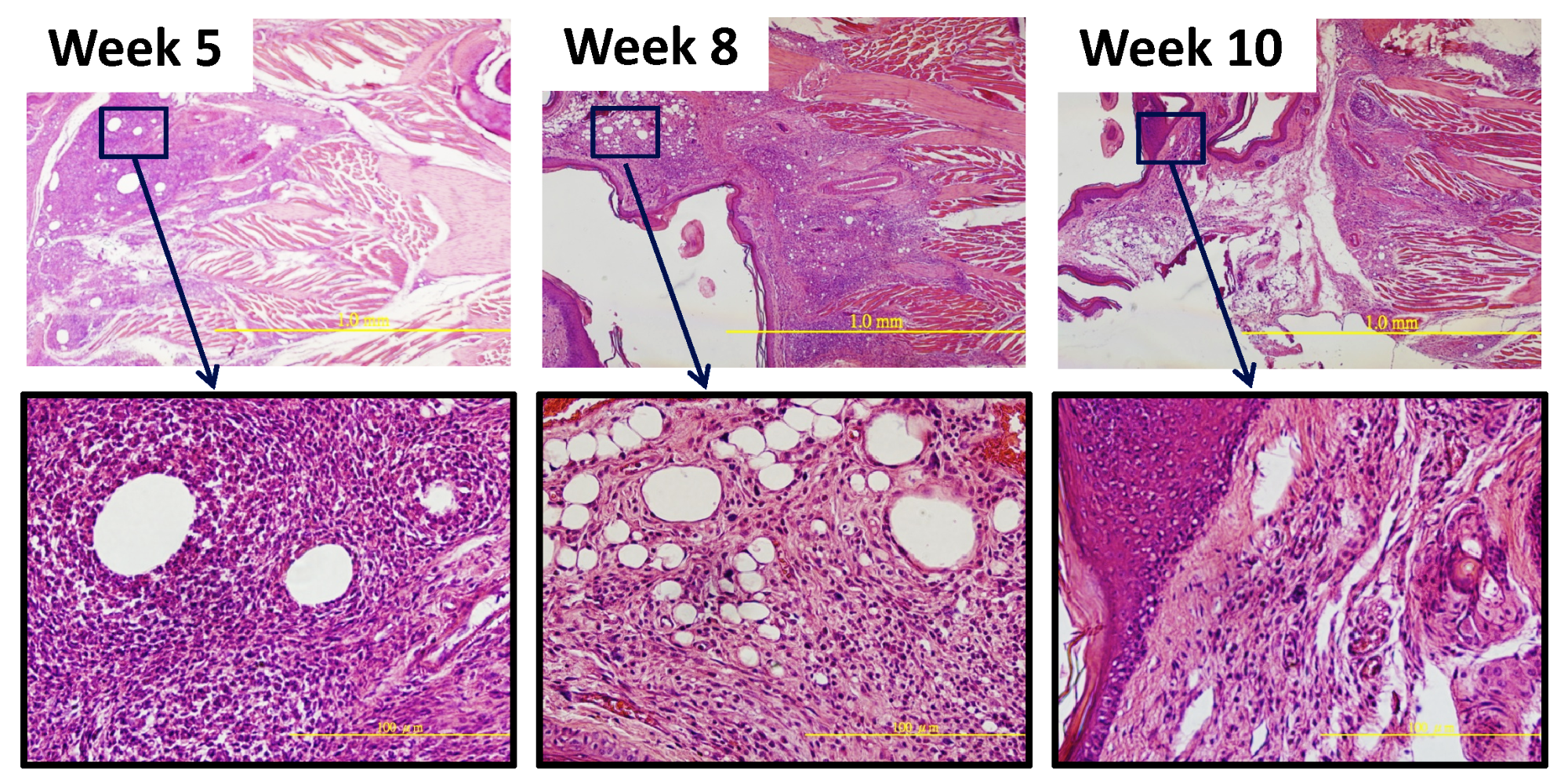


**Figure S3.** H&E staining images of the injection site at different time points after s.c. footpad injection of PELC-formulated OVA. (original magnification, ×100 and ×400). Cells infiltrated all around the vacuoles (identified as PELC emulsion) at the injection sites within 5 weeks post-injection. The vacuoles became smaller over time, and the infiltrated cells were retained for up to 10 weeks.


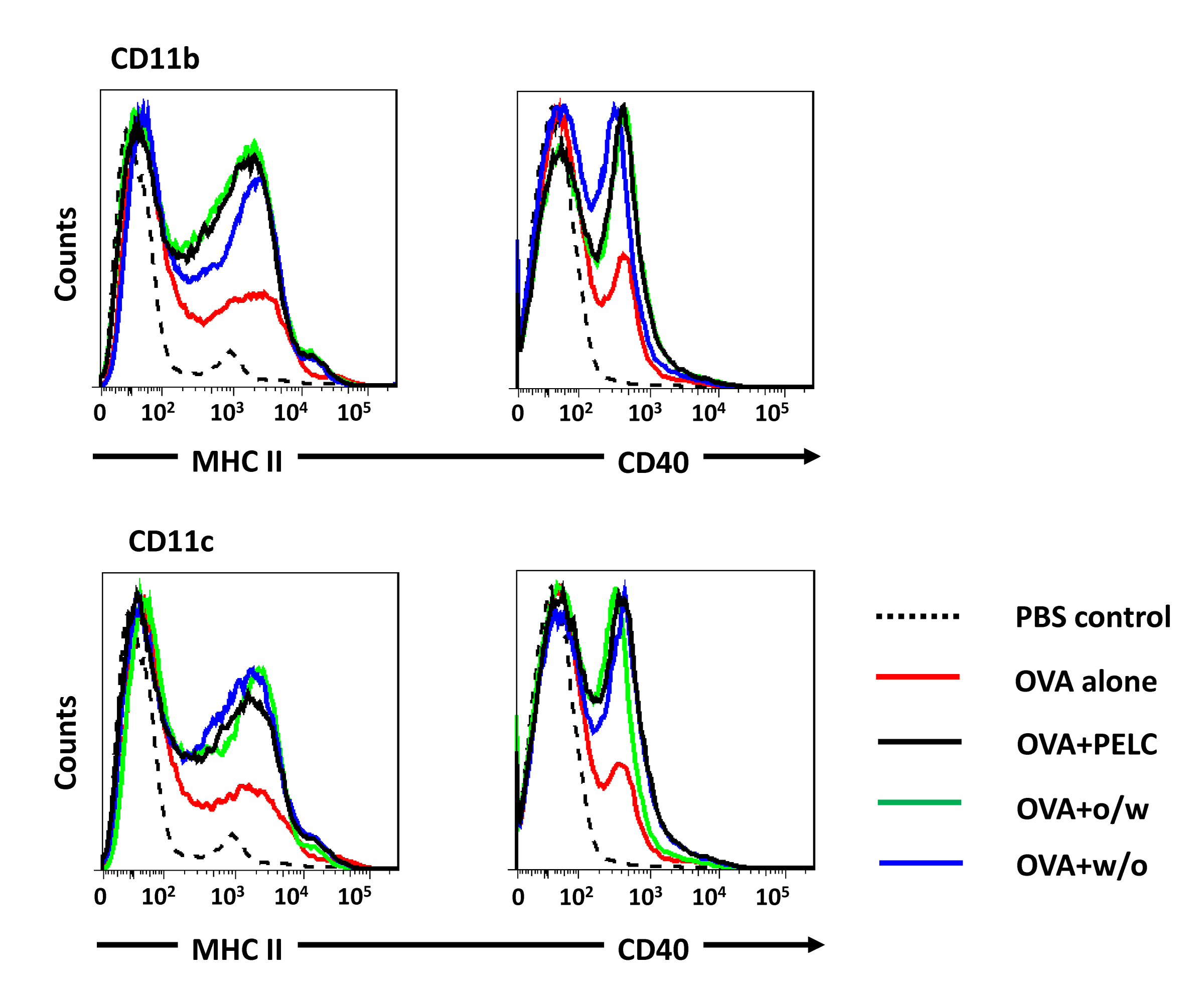


**Figure S4.** Activation of the draining LN cells. C57BL/6 mice (3 mice/group) were injected once s.c. in both hind footpads with 10 μg/mL of OVA, alone or formulated with various adjuvant candidates. The draining LN cells were harvested at day 7, and the expression levels of MHC class II and CD40 were determined by flow cytometry. The flow cytometric histograms shown were gated on CD11b^+^ or CD11c^+^ cells. The results are representative of two independent experiments.


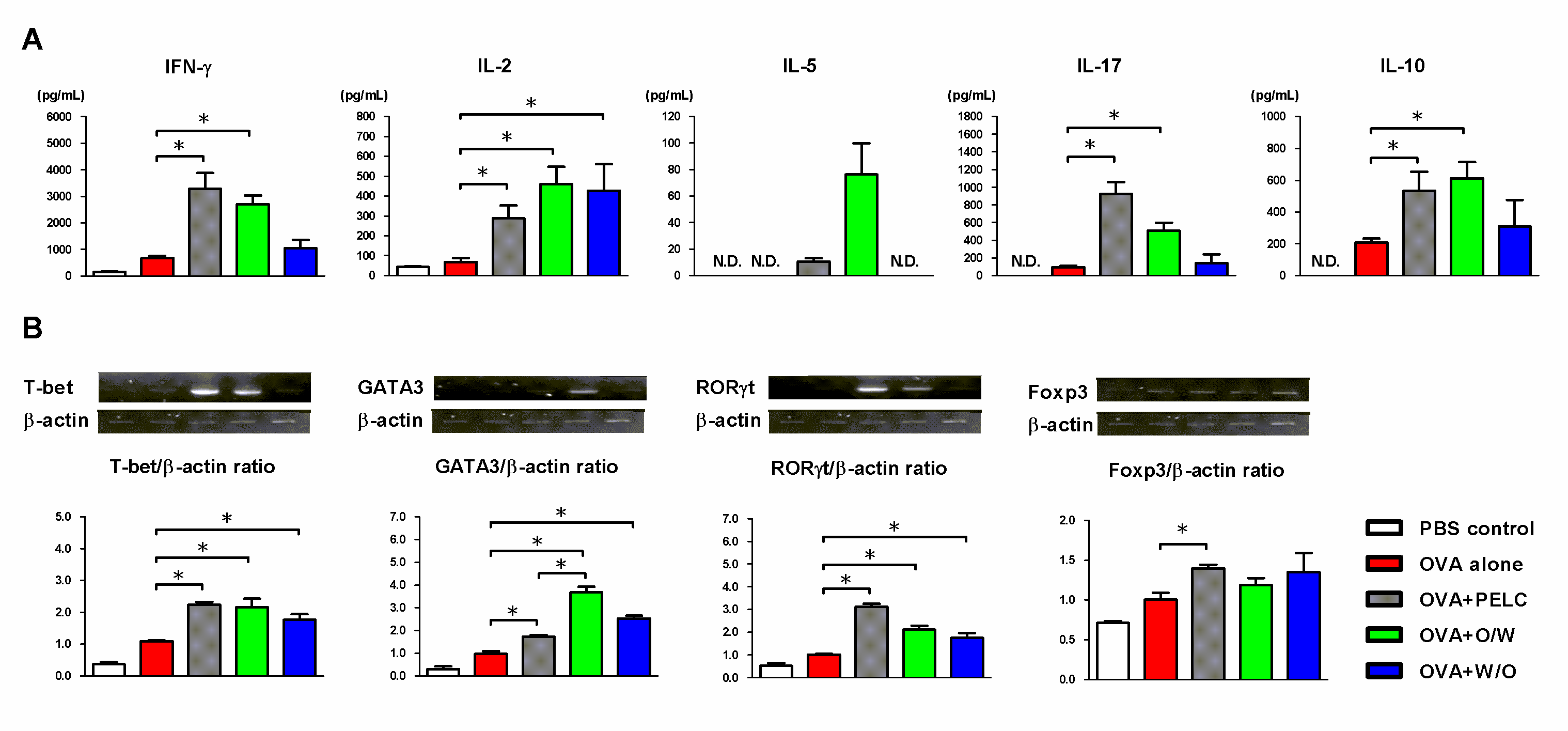


**Figure S5.** Analysis of T cell immunity. C57BL/6 mice (3 mice/group) were injected once s.c. in both hind footpads with 10 μg/mL OVA alone or OVA formulated with various adjuvant candidates. Seven days after the vaccination, splenocyte suspensions (5×10^6^ cells/mL) were pooled and incubated in the presence or absence of 50 μg/mL OVA protein for 72 h. (A) Supernatants from triplicate cultures were collected to measure the concentrations of cytokines IFN-γ, IL-2, IL-5, IL-17 and IL-10 by ELISA via paired antibodies. The data are presented as cytokine release in the presence of OVA minus release in the presence of medium only. (B) The mRNA expression levels of T-bet, GATA3, RORγt and Foxp3 were measured by RT-PCR. The data are normalized to the β-actin mRNA and graphed as the fold change over the non-adjuvanted OVA control. **P* < 0.05. The data are expressed as the mean plus the standard errors of triplicate assays. The results are representative of two independent experiments.


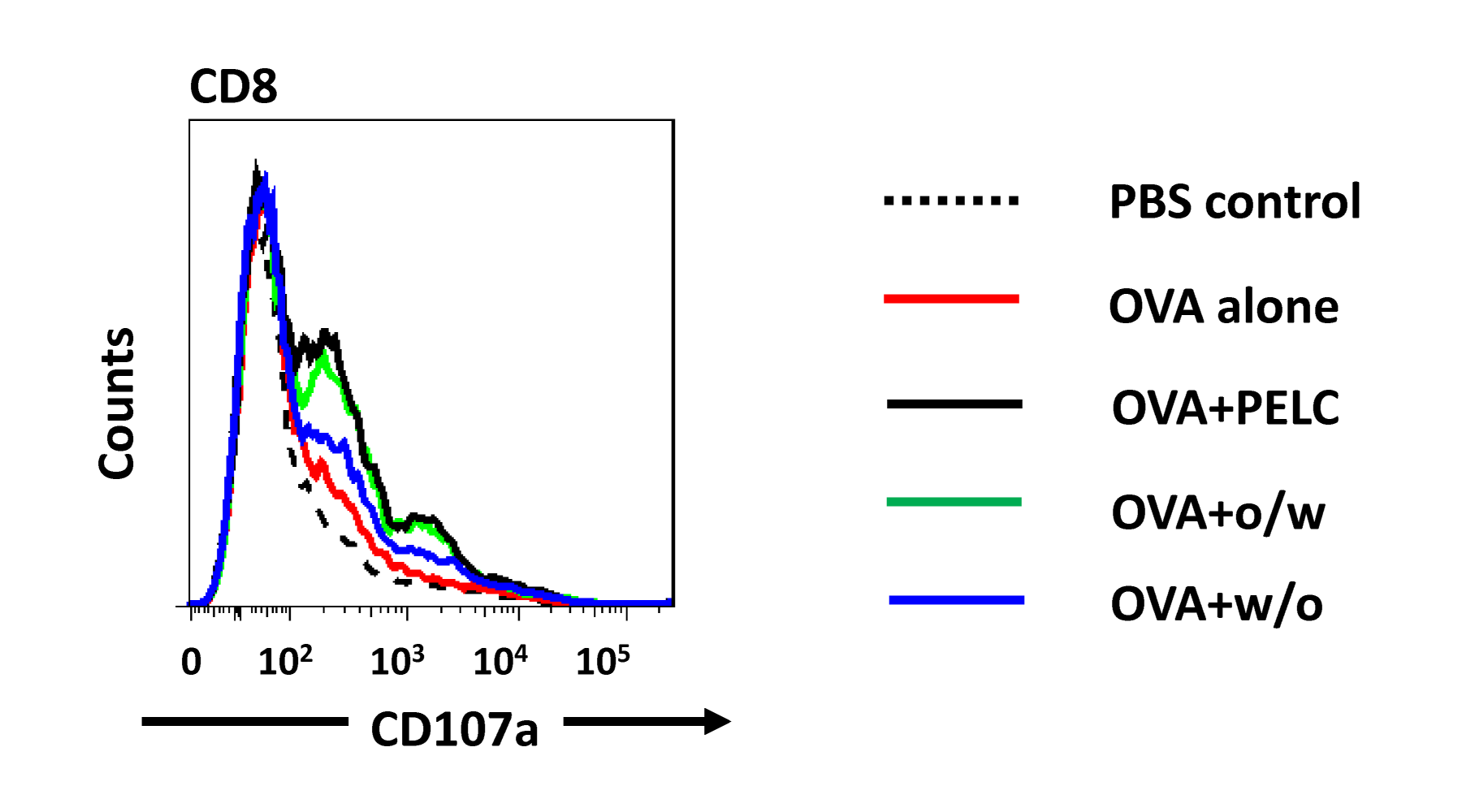


**Figure S6.** Activation of CD8^+^ splenic T cells. C57BL/6 mice (3 mice/group) were injected once s.c. in both hind footpads with 10 μg/mL OVA alone or OVA formulated with various adjuvant candidates. Seven days after the vaccination, splenocyte suspensions (5×10^6^ cells/mL) were pooled and incubated in the presence or absence of 50 μg/mL OVA protein for 72 h. The expression level of CD107a was determined by flow cytometry. The data shown were gated on CD8^+^ T cells. The results are representative of two independent experiments.

**Experimental Section**

**Histological examination and serum biochemistry test.** For the histological examination, C57BL/6 mice (six mice per group) were injected i.m. in the quadriceps with 200 μl of PBS alone (negative control group) or supplemented with 20 μg of PELC or a water-in-oil emulsion of PBS in incomplete Freund's adjuvant (IFA; Sigma) at a PBS/IFA ratio of 1/1 v/v as a positive control group. On day 14 post-injection, the tissues of the injection site were excised, sectioned, and stained with hematoxylin and eosin (H&E) by the Pathology Core Laboratory of NHRI for histological examination. For the biochemistry test, C57BL/6 mice (six mice per group) were injected once i.m. with 0, 10, 50, or 100 % v/v PELC diluted in PBS. Serum samples were collected at weeks 0, 2, 4, 8, and 12 via the submandibular veins and analyzed in the Laboratory Animal Center of NHRI for the measurement of AST, ALT, and CRE. Regarding the bioresorption study, C57BL/6 mice injected s.c. with 50 μl of 10 % v/v PELC in PBS in both hind footpads were sacrificed on weeks 5, 8 and 10 post-injection. The footpad tissues were excised, sectioned, and stained with H&E for histological examination.

**Supplementary Text**

**Safety aspects of PELC.** To investigate whether PELC was harmful to the host, a histological examination of the injection site tissues was conducted (Figure S2A). The PBS group served as the negative control, and incomplete Freund's adjuvant (IFA, W/O emulsion) was administered to the positive control group. No cellular infiltration was observed in the PBS group after injection, whereas cellular infiltration was widespread and severe necrosis of the adipose tissue was observed at the local injection sites in the IFA group. Recruitment of infiltrated cells to the injection sites was observed in the PELC group; however, cell necrosis and calcification were rarely visible around the injected mass (Figure S2A). These findings indicate that injection with PELC induced less tissue damage compared to IFA. Because IFA causes very extensive tissue inflammation, the adjuvant use of IFA is restricted to research purposes in laboratory animals. The low squalene oil content (<5% v/v) in the PELC-formulated vaccine causes only mild inflammatory reactions and is prospectively used as an adjuvant in vaccines for human use. Serum samples from the mice treated with PELC emulsion were further calorimetrically analyzed for liver and kidney functions. The results showed that biochemical indicators, including aspartate aminotransferase (AST), alanine aminotransferase (ALT), and creatinine (CRE), were in the normal range (AST: 70-400 IU/L; ALT: 25-100 IU/L; and CRE: 0.2-0.6 mg/dL) and no differences were observed between groups (Figure S2B). Furthermore, no obvious clinical signs of autoimmune or allergic disorders were observed in the mice during the 40 weeks following PELC injection. In summary, mice show considerable tolerance for PELC, which is a feature of great interest for *in vivo* applications.

**PELC reshapes cell-mediated immunity *in vivo*.** To determine the impact of candidate compounds on the immune cells, we vaccinated mice with OVA, alone or formulated with PELC, an O/W-type emulsion (AddaVax^TM^, a squalene-based oil-in-water emulsion based on the formulation of MF59; InvivoGen), or a W/O-type emulsion (IFA) through footpad injection, followed by measuring the level of activation marker expression on CD11b^+^ cells harvested from the draining LNs. As shown in Figure S4A, vaccination of O/W-adjuvanted OVA enhances the expression of MHC class II and CD40, as compared with OVA alone. However, administration of W/O-adjuvanted OVA lowers the level of activation marker expression as that induced by O/W-adjuvanted OVA. PELC retains the ability of O/W to modulate the activation of the immature CD11b^+^ cells. Similar results were observed with CD11c^+^ cells post-injection (Figure S4B). OVA formulated with PELC or O/W emulsion enhance effectively the activation of CD11c^+^ cells. Nevertheless, the potency was rather reduced when OVA was co-administrated with the W/O emulsion. Collectively, both PELC and O/W could induce phenotypic changes on the draining LNs.

T cell cytokine responses were measured in the spleen following re-stimulation of the cells *in vitro* with the OVA antigen. Figure S5A showed that following vaccination, W/O-adjuvanted OVA with did not conspicuously induce antigen-specific cytokine production, such that the IFN-γ, IL-5, IL-10, and IL-17 concentrations were at the same level as those induced by non-adjuvanted OVA, indicating W/O probably could not be an adjuvant to elicit appropriate cellular response. OVA adjuvanted with O/W emulsion did enhance a notable cellular response, and the IFN-γ, IL-2, IL-5, IL-10, and IL-17 concentrations detected in the splenocyte supernatants were significantly higher than those in the non-adjuvanted group. Interestingly, sufficiently elevated IFN-γ and IL-17 secretion was detected in splenocyte supernatants collected from mice treated with PELC emulsion, whereas the T_H_2-type cytokines IL-5 and IL-10 were at the reduced level compared with the level in the O/W-adjuvant group (Figure S5A). The mRNA expression of transcription factors, including T-bet (T_H_1), GATA3 (T_H_2), RORγt (T_H_17) and Foxp3 (T_reg_), was characterized in both T cell subsets to assess T cell differentiation (Figure S5B). Vaccination with OVA adjuvanted with O/W emulsion augmented T-bet, GATA3 and RORγt mRNA expression compared with vaccination with OVA alone. Nevertheless, vaccination with PELC-adjuvanted OVA increased mRNA expression of T-bet and RORγt but diminished GATA3 mRNA expression compared with vaccination of OVA without the formulation. General speaking, vaccination with W/O-adjuvanted OVA never induced important mRNA expression. The above results indicate that immunization with PELC shifted T cell immunity toward T_H_1 and T_H_17 polarization but diminished T_H_2 immune responses. The functional activation of the T cells induced by emulsions was also confirmed using CTL phenotyping (Figure S6). The data substantiated that the PELC emulsion is capable of stimulating the antigen-specific CTL activity of cells from the plenocytes, in agreement with the findings from cytokine secretion. Interestingly, it appears that mice treated with PELC adjuvant showed the strongest OVA-specific immune response among the three adjuvants. Together, PELC emulsion has the intermediate mechanisms between the particulate depot and immunomodulator in terms of magnitude of the induced activation of draining LNs and T cells, compared with O/W and W/O emulsions.
